# Supplementary material for: Pyruvate Kinase Deficiency: Markedly Decreased Reticulocyte PK Activity and Limited Specificity of the PK/HK Ratio
Source: Int J Mol Sci. 2025 Sep 4;26(17):8606. doi: 10.3390/ijms26178606 (PMC12429783; doi:10.3390/ijms26178606)
Supplement: Supplementary file 1 [file ijms-26-08606-s001.zip › ijms-3827990-supplementary.pdf]

Pyruvate kinase deficiency: markedly decreased reticulocyte PK activity and limited specificity of the PK:HK ratio

SUPPLEMENTARY DATA

Results

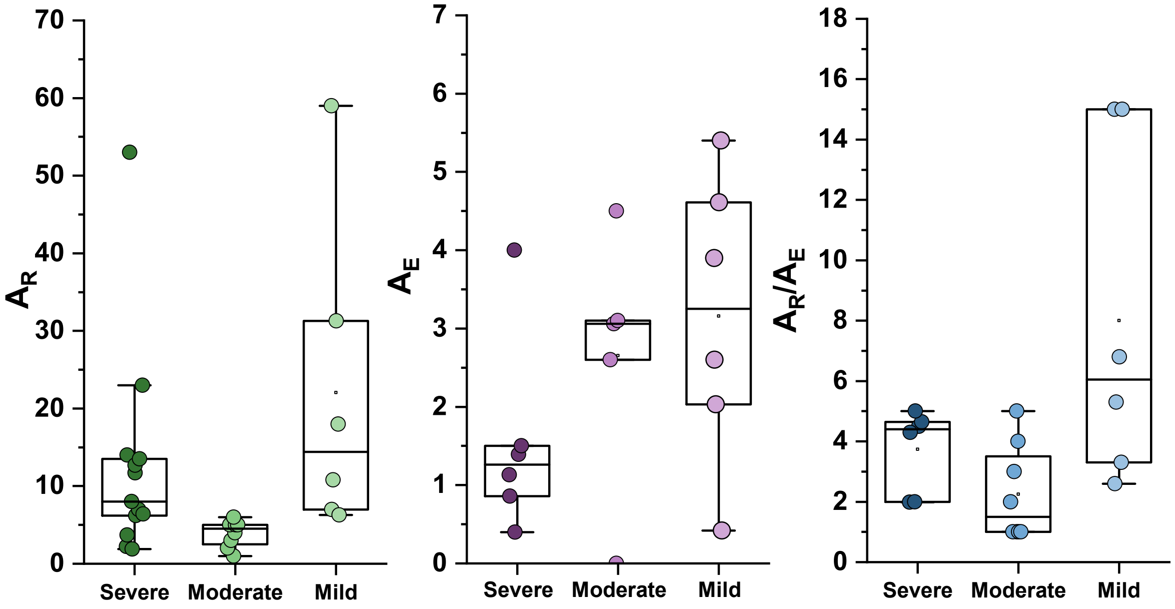

Figure S1. Dependence of specific activity in reticulocytes (A_R) and erythrocytes (A_E) and their ratio (A_R/ A_E) on the severity of anemia. Patients were distributed into different groups by severity depending on the frequency of donor erythrocytes transfusions. No significant differences were found between groups for each parameter.

Characteristics of patients included in the study

Data on all patients included in the study are presented in Table S1.

Table S1. Characteristics of patients enrolled in the study

Patient	Age	Diagnosis	Mutated gene	cDNA nucleotide substitution allele1/allele2	Sex	Age at anemia diagnosis	Frequency of donor erythrocyte transfusions	Splenectomy	Hb g/l	RET, %	Unconjugated bilirubin, µmol/l
**Pyruvate kinase deficiency patients**											
Severe condition											
1. *	6 y	Pyruvate kinase deficiency of RBCs	*PKLR*	homo c.101-1G>A	F	From birth	Monthly	No	88	2.19	61,9
2.	7 y	Pyruvate kinase deficiency of RBCs	*PKLR*	homo c.401T>A	F	From 3 months	Monthly	At 2 y.o.	74	41.19	57.9
3.	11 y	Pyruvate kinase deficiency of RBCs	*PKLR*	homo c.695-2A>C	M	From birth	Monthly up to 3 y. o., then 1-2 times a year	Selective, at 4 y.o.	78	28.93	n/d
4. *	17 y	Pyruvate kinase deficiency of RBCs	*PKLR*	homo c.1079G>A	M	From birth	Monthly	No	86	3.1	120
5. *	6 y	Pyruvate kinase deficiency of RBCs	*PKLR*	homo c.1269+1G>A	F	From birth	Monthly	No	68	2.98	134
6. *	19 y	Pyruvate kinase deficiency of RBCs	*PKLR*	homo c.1529G>A	F	From birth	Every 3 months up to 11 y. o., then 2-3 times a year	No	97	3.65	18.7
7. *	2 y	Pyruvate kinase	*PKLR*	homo c.1529G>A	M	From birth	1-2 times a month up to 4 months	No	114	5.56	30.5

		deficiency of RBCs									
8.	2 y	Pyruvate kinase deficiency of RBCs	*PKLR*	homo c.1529G>A	F	From birth	Monthly up to 9 months	No	98	6.70	21,5
9.	18 y	Pyruvate kinase deficiency of RBCs	*PKLR*	homo c.1529G>A	M	From 2 month	Every 1.5-2 months in the first 2 years of life	At 14 y.o.	110	16.47	151,5
10.	12 y	Pyruvate kinase deficiency of RBCs	*PKLR*	homo c.1529G>A	F	From birth	Monthly in the first years of life, then n/d	No	63	11.45	76,5
11.	11 m	Pyruvate kinase deficiency of RBCs	*PKLR*	homo c.1529G>A	F	From birth	4 times in the first years of life, then n/d	No	66	20.77	49,4
12. *	26 y	Pyruvate kinase deficiency of RBCs	*PKLR*	c.101-1G>A/c.1318G>T	F	From birth	Monthly	No	n/d	n/d	n/d
13. *	3 y	Pyruvate kinase deficiency of RBCs	*PKLR*	c.948C>A/c.848T>A	F	n/d	Monthly	n/d	79	1.8	34
14.	8 y	Pyruvate kinase deficiency of RBCs	*PKLR*	c.1130T>C/c.1318G>T	F	From birth	Monthly up to 7 y. o., then once a year	At 7 y.o.	n/d	n/d	n/d
15. *	5 y	Pyruvate kinase deficiency of RBCs	*PKLR*	c.1174G>A/c.1456C>T	M	From birth	Every 1.5-2 months	No	76	25.22	n/d
16. *	3 y	Pyruvate kinase deficiency of RBCs	*PKLR*	c.1174G>A/c.1456C>T	M	From birth	Monthly	No	69	4.31	101.5

17. *	6 y	Pyruvate kinase deficiency of RBCs	*PKLR*	c.1436G>A/c.487C>T	F	From birth	Every 3 months	No	n/d	n/d	n/d
18.	4 y	Pyruvate kinase deficiency of RBCs	*PKLR*	c.1456C>T/c.1157C>T	F	From 1 month	Every 1-2 months up to 10 months	No	94	7.36	31,6
19. *	15 y	Pyruvate kinase deficiency of RBCs	*PKLR*	c.1529G>A/Ex 1-2 del	F	From birth	Monthly up to 11 years, after that once every 3 months	At 11 y.o.	76	66.24	47.7
20. *	9 y	Pyruvate kinase deficiency of RBCs	*PKLR*	c.1529G>A/Ex 1-2 del	M	From birth	Every 2-3 months up to 9 y. o.	At 9 y.o.	87	31.37	58.6
21. *	15 y	Pyruvate kinase deficiency of RBCs	*PKLR*	c.1529G>A/Ex 1-2 del	F	From birth	Regularly up to 15 y. o.	At 15 y.o.	96	n/d	77,5
22. *	3 y	Pyruvate kinase deficiency of RBCs	*PKLR*	c.1529G>A/c.-63G>A	M	From 2 month	Every 1.5 months	No	87	4.89	94.5
23. *	6 y	Pyruvate kinase deficiency of RBCs	*PKLR*	c.1529G>A /c.101-1G>A	M	From birth	Every 2 months	No	71	8.83	58,1
24. *	2 y 8 m	Pyruvate kinase deficiency of RBCs	*PKLR*	c.1529G>A/c.460G>A	F	From birth	Every 2-3 months	No	86	6.07	42.1
25. *	11 y	Pyruvate kinase deficiency of RBCs	*PKLR*	c.1529G>A/c.994G>A	M	From birth	Monthly	No	75	5.97	n/d
26.	12 y	Pyruvate kinase	*PKLR*	c.1529G>A/c.1079G>A	M	From birth	Monthly up to 5 y. o.	At 5 y.o.	85	24	59.5

		deficiency of RBCs									
27.	23 y	Pyruvate kinase deficiency of RBCs	*PKLR*	c.1529G>A/c.1223C>T	F	From birth	Transfusion dependence up to 9 y. o.	At 9 y.o.	n/d	n/d	n/d
28. *	11 y	Pyruvate kinase deficiency of RBCs	*PKLR*	c.1583A>T/c.1436G>A	F	From birth	Every 2-3 weeks	No	76	4.8	n/d
29. *	14 y	Pyruvate kinase deficiency of RBCs	*PKLR*	c.1637T>C/c.1529G>A	F	From birth	Every 2 months	No	44	24.44	179.3
Moderate condition											
30.	9 y	Pyruvate kinase deficiency of RBCs	*PKLR*	homo c.1318G>A	M	From birth	Once a year	No	74	15.51	69.2
31.	2 y	Pyruvate kinase deficiency of RBCs	*PKLR*	c.932T>C/c.1456C>T	M	From 2-3 months	4 transfusions in the 1st year of life	No	101	3.96	15.2
32.	10 y	Pyruvate kinase deficiency of RBCs	*PKLR*	c.1231G>T/c.1456C>T	F	From birth	Several times in the first year of life	No	90	7.62	78.2
33.	13 y	Pyruvate kinase deficiency of RBCs	*PKLR*	c.1231G>T/c.1456C>T	F	From birth	About 6 transfusions up to 7 y. o.	No	84	10.94	171.2
34. *	5 m	Pyruvate kinase deficiency of RBCs	*PKLR*	c.1456C>T/c.1130T>C	M	From birth	3 times in the first six months of life	No	94	5.43	18.5
35. *	13 m	Pyruvate kinase deficiency of RBCs	*PKLR*	c.1456C>T/c.1594C>T	F	From 1 month	Once, at 1 month	No	102	2.91	n/d

36.	8 y	Pyruvate kinase deficiency of RBCs	*PKLR*	c.1130T>C/c.1456C>T	F	From birth	Several times in the first year of life	At 8 y.o.	92	6.09	n/d
Mild condition											
37.	14 y	Pyruvate kinase deficiency of RBCs	*PKLR*	c.932T>C/c.1456C>T	M	From 1 month	Once in the first month of life	No	84	n/d	n/d
38.	10 y	Pyruvate kinase deficiency of RBCs	*PKLR*	c.1076G>A/c.1456C>T	F	From 6 months	No transfusions	No	101	7.45	192,1
39.	7 y	Pyruvate kinase deficiency of RBCs	*PKLR*	c.1181C>T/c.1456C>T	F	From 5,5 months	2 transfusions per life	No	92	4.89	17
40.	5 y	Pyruvate kinase deficiency of RBCs	*PKLR*	c.1181C>T/c.1456C>T	M	From birth	2 times in the first 2 months of life	No	82	6.58	31.9
41.	17 y	Pyruvate kinase deficiency of RBCs	*PKLR*	c.1195del /c.1456C>T	M	From 1 year 4 months	No transfusions	No	124	5.11	72.8
42.	12 y	Pyruvate kinase deficiency of RBCs	*PKLR*	c.1291G>A/c.1529G>A	M	From 5 years	No transfusions	No	118	4.52	53
43.	8 y	Pyruvate kinase deficiency of RBCs	*PKLR*	c.1429A>G/c.665G>A	F	From 3 months	No transfusions	No	97	6.03	34.4
44.	14 y	Pyruvate kinase deficiency of RBCs	*PKLR*	c.1529G>A/c.1072G>T	F	From 1 year	2 transfusions per life	No	82	18.9	75.6

45.	9 y	Pyruvate kinase deficiency of RBCs	*PKLR*	c.1583A>T/c.1510C>T	F	From birth	Once, at 3 y. o.	No	105	4.06	156,6
46.	19 y	Pyruvate kinase deficiency of RBCs	*PKLR*	homo c.1529G>A	M	n/d	n/d	n/d	101	4.3	124.1
**Patients with other anemias**											
47.	2 y	Hereditary hemolytic anemia, unspecified	*ALAD*	het c.375del	M	From 3 months	No transfusions	No	106	n/d	n/d
48.	5 y	Hereditary spherocytosis, type 1	*ANK1*	het c.5097-33G>A	M	From birth	No transfusions	No	108	6.86	16
49.	5 y	Hereditary spherocytosis, type 1	*ANK1*	het c.596dup frameshift ter	F	From birth	6 transfusions in life	n/d	113	17.21	30.2
50.	16 y	Hereditary hemolytic anemia, unspecified	*ANK1*	het c.4104+4A>G	F	From 1 month	Every six months	At 15 y.o.	125	3.02	8.3
			PIEZO1	het c.3284A>C							
51.	6 y	Hereditary spherocytosis, type 1	*ANK1*	het c.3329_3336 delinsACAAG	M	From 6 years	No transfusions	No	104	4.62	27.2
52. *	13 y	Hereditary spherocytosis, type 1	*ANK1*	het c.3778T>C	F	--	Once in life	No	126	3.86	29
53. *	8 m	Hereditary spherocytosis, type 1	*ANK1*	het c.1814del	M	From birth	Every 3-4 weeks	No	104	6.6	19.4
54.	11 y	Hereditary spherocytosis, type 1	*ANK1*	het c.596dup frameshift ter	F	From birth	Once at 11 years old	No	107	10.08	27.7

55. *	2 m	Hereditary spherocytosis, type 1	*ANK1*	het c.4153C>T	M	From birth	Every week	No	84	4.2	17.5
56.	4 m	Hereditary spherocytosis, type 1	*ANK1*	het c.2325dupG	F	n/d	No transfusions	No	n/d	n/d	n/d
57.	7 y	Glucose phosphate isomerase deficiency of RBCs	*GPI*	c.1039C>T/c.1612C>A	M	From 3 years	Transfusion dependent	At 6 y.o.	94	18.38	80.7
58. *	7 m	Beta thalassemia	*HBB*	het c.193G>T	F	From birth	Monthly	No	93	0.1	n/d
59. *	7 m	Hexokinase deficiency of RBCs	*HK1*	c.1951G>A/c.2128G>A	F	From birth	Every 2-3 weeks	No	76	6.33	n/d
60. *	5 m	Hexokinase deficiency of RBCs and Beta thalassemia	*HK1*<br>*HBB*	homo c.34C>T<br>homo c.316-106 C>G	M	From birth	Monthly	No	83	0.54	n/d
61.	9 y	Dehydrated hereditary stomatocytosis, type 2	*KCNN4*	het c.940T>C	M	From 1st year of life	No transfusions	No	102	6.54	191.6
62.	16 y	Dehydrated hereditary stomatocytosis	*PIEZO1*	het c.7483_7488dup	M	From 11 years	No transfusions	No	102	6.08	16
63.	12 y	Dehydrated hereditary stomatocytosis	*PIEZO1*	het c.7483_7489dup	M	From birth	n/d	No	114	11.21	105.2
64.	17 y	Paroxysmal nocturnal hemoglobinuria 1	*PIGA*	c.264delA/c.715+1G>A	F	From 14 years	No transfusions	No	88	12.31	9.3
65. *	10 y	Diamond-Blackfan anemia, type 7	*RPL11*	het c.45delT	M	n/d	Every 2-3 months	No	87	1.16	n/d

66. *	8 m	Diamond-Blackfan anemia, type 1	*RPS19*	het c.3G>A	F	From 2 months	Every 3 weeks	No	78	0.69	n/d
67.	15 y	Hereditary spherocytosis, type 4	*SLC4A1*	het c.1030C>T	M	From 15 years	No transfusions	No	130	7.23	59.7
68.	15 y	Hereditary spherocytosis, type 4	*SLC4A1*	het c.1030C>T	M	n/d	No transfusions	No	130	5.2	42.5
69.	17 y	Hereditary spherocytosis, type 4 or Southeast Asian ovalocytosis or Cryohydrocytosis	*SLC4A1*	het c.749del	M	From 4 years	No transfusions	No	135	7.4	n/d
70.	9 y	Hereditary elliptocytosis, type 2	*SPTA1*	het c.82C>T	F	From birth	Every 2-3 months up to 5 y. o.	No	115	6.33	43.9
71.	8 y	Hereditary elliptocytosis, type 2	*SPTA1*	het c.82C>T	M	From 3 months	Irregular, 3 transfusions per life	No	103	11.61	31.4
72.	8 y	Hereditary elliptocytosis, type 2	*SPTA1*<br>*NF1*<br>*KDM6A*	het c.4019C>A<br>het c.4614G>A<br>hemi c.2308C>T	M	From 1 year	Once, at 2 years old	No	125	1.33	n/d
73.	8 y	Hereditary elliptocytosis, type 2	*SPTA1*<br>*FLNA*<br>*COL3A1*<br>*CFH*	het c.5645_5647delGAG<br>het c.1790_1791delTT<br>het c.689A>T<br>het c.2407T>A	F	From 2 years	Irregular, 8 transfusions per life	No	128	2.37	5.8
74.	11 y	Hereditary spherocytosis, type 3	*SPTA1*	c.4339-99C>T/c.6421C>T	F	From birth	No transfusions	No	99	9.56	60.8
75.	8 y	Hereditary elliptocytosis, type 2 and Beta talassemia	*SPTA1*<br>*HBB*	het c.2671C>T<br>het c.364G>C	M	From 1 year 6 months	3 transfusions at 8 years old	No	92	11.95	36.6

76.	1 y	Hereditary elliptocytosis, type 3	*SPTB*	het c.566+1G>A	F	From birth	Monthly	No	88	12.34	n/d
77.	9 y	Hereditary spherocytosis, type 2	*SPTB*	het c.1912C>T	F	From 3 months	Once, at 8 y. o.	No	99	16.82	35.7
			PKLR	het c.1456C>T							
78.	9 y	Elliptocytosis, type 3 or Spherocytosis, type 2	*SPTB*	het c.5800_5801insCAGG	F	From 9 years	Once, at 9 y. o.	No	93	13	60
79.	3 y	Hereditary spherocytosis, type 1	*ANK1*	het c.4462C>T	F	From 3 years	No transfusions	No	96	9.25	19.3
80.	51 y	Myelodysplastic syndrome	*SF3B1*	n/d	F	n/d	n/d	n/d	70	n/d	n/d
81.	13 y	Hereditary elliptocytosis, type 2	*SPTB*	het c.2491C>T	M	From 1 month	Once, at 1 month	No	121	5.58	62.9
82. *	2 y	Hemolytic anemia, unspecified	*CASP10*	het c.1316 G>A	F	From 1 year	Against the background of infections 3 transfusions	No	85	7.14	38.4
83. *	21 y	X-linked sideroblastic anemia, type 1	*ALAS2*	hemi c.484T>C	M	From 3 years	10 transfusions at 21 y. o.	No	108	n/d	16.3
84.	5 y	Hereditary spherocytosis, type 1	*ANK1*	het c.5167G>T	F	From birth	Every 2-3 months	No	92	6.18	18.2
85. *	3 m	Hereditary spherocytosis, type 1	*ANK1*	het c.3754C>T	M	From 1 month	Monthly	No	101	1.5	n/d
86.	8 y	Hereditary spherocytosis, type 1	*ANK1*	het c.5097-33G>A	F	From 3 years	No transfusions	No	111	3.06	9.7

87.	11 y	Hereditary spherocytosis, type 1	*ANK1*	het c.5097-33G>A	F	From 5 years	No transfusions	No	95	16.88	76
88. *	11 y	Hereditary spherocytosis, type 1	*ANK1*	het c.3630-2A>C	M	From 11 years	Once, 1 month before the analysis	No	105	6.21	n/d
89.	13 y	Megaloblastic anemia, type 1	*CUBN*	het c.4426C>T	F	From birth	Irregularly, several times in life	No	102	7.28	100
90.	6 m	*G6PD* - deficiency of RBCs	*G6PD*	hemi c.686A>C	M	From 7 months	No transfusions	No	106	4.88	n/d
91. *	8 y	*G6PD* - deficiency of RBCs	*G6PD*	hemi c.1178G>A	M	From birth	Once, at 8 y. o.	No	115	4.36	23.9
92.	7 y	Alpha-thalassemia	*HBA2*	het chr16:173000-176000 del of gene HBA2	M	From 4 years	Once, at 4 y. o.	No	123	0.75	4.1
93.	10 мес	Beta-thalassemia	*HBB*	homo c.92+5G>C	M	From 4 months	No transfusions	No	129	n/d	n/d
94.	4 y	Dehydrated hereditary stomatocytosis, type 2	*KCNN4*	het c.1055G>A	M	From birth	Once, at 1 month	No	102	8.86	34.5
95.	19 y	Dehydrated hereditary stomatocytosis or xerocytosis	*PIEZO1*	het c.7285C>T	F	From 8 years	No transfusions	n/d	90	7.31	n/d
96.	24 y	Congenital dyserythropoietic anemia, type 2	*SEC23B*	c.279+5G>A/c.325G>A	F	n/d	n/d	n/d	n/d	n/d	n/d
97.	17 y	Atypical hemolytic	*SLC19A1*	het c.634_636del	F	From 5 years	No transfusions	No	112	5.56	26.3

		uremic syndrome, type 7 or Congenital dyserythropoietic anemia, type 2	*SEC23B*	het c.970C>T							
			DGKE	het c.610dup							
98.	61 y	Hereditary elliptocytosis, type 2	*SPTA1*	het c.4462C>T	M	From 43 years	n/d	n/d	121	6.81	n/d
99.	11 y	Hereditary spherocytosis, type 3	*SPTA1*	het c.2222A>T	F	From 3 years	No transfusions	At 5 y.o.	138	2.89	8.9
100.	14 y	Hereditary spherocytosis, type 3	*SPTA1*	het c.3996G>T	F	From 13 years	No transfusions	At 5 y.o.	108	n/d	80.6
101.	3 y	Hereditary spherocytosis, type 2	*SPTB*	het c.3697A>T	M	From birth	Once, at 2 months	No	106	10.17	n/d
102.	17 y	Hereditary spherocytosis, type 2	*SPTB*	het c.300+2T>C	F	From birth	No transfusions	At 6 y.o.	119	2.91	14.3
103.	12 y	Hereditary spherocytosis, type 2	*SPTB*	het c.1064+1G>A	F	From 12 years	No transfusions	No	101	7.35	43.5
104.	11 y	Hereditary spherocytosis, type 2	*SPTB*	het c.332A>G	M	From birth	No transfusions	No	123	9.43	n/d
105.	4 y	Hereditary spherocytosis, type 2	*SPTB*	het c.2282_2283del	F	From 4 months	2 transfusions per life	No	82	13.4	n/d
106.	16 y	Hereditary spherocytosis, type 2	*SPTB*	het c.2176del	F	From 15 years	No transfusions	No	125	9.5	34.7
107.	2 m	Hereditary spherocytosis, type 2	*SPTB*	het c.3082C>T	M	From birth	Monthly	No	90	2.74	9.1
108.	9 y	Hereditary elliptocytosis, type 3	*SPTB*	het c.3561+1G>A	F	n/d	No transfusions	No	123	4.91	66.6

*- Patients with recent transfusions (≤ 3 months prior to PK activity analysis); y –year; m –month; y. o. – years old; RBCs – red blood cells; F- female; M – male; het -heterozygous mutation; homo – homozygous mutation; hemi – hemizygous mutation; *G6PD* - glucose-6-phosphate dehydrogenase; RET - reticulocyte count in the blood in %; Hb - hemoglobin concentration in the blood in g/l; n/d – not determined. Blood parameters such as Hb, RET, unconjugated bilirubin are given on the date closest to the PK activity analysis. Patients were distributed into different groups by severity depending on the frequency of donor erythrocytes transfusions
